# Preferential Targeting of Na_v_1.6 Voltage-Gated Na^+^ Channels to the Axon Initial Segment during Development

**DOI:** 10.1371/journal.pone.0124397

**Published:** 2015-04-15

**Authors:** Elizabeth J. Akin, Laura Solé, Sulayman D. Dib-Hajj, Stephen G. Waxman, Michael M. Tamkun

**Affiliations:** 1 Cell and Molecular Biology Graduate Program, Colorado State University, Fort Collins, Colorado, United States of America; 2 Department of Biomedical Sciences, Colorado State University, Fort Collins, Colorado, United States of America; 3 Department of Biochemistry and Molecular Biology, Colorado State University, Fort Collins, Colorado, United States of America; 4 Molecular, Cellular and Integrative Neuroscience Program, Colorado State University, Fort Collins, Colorado, United States of America; 5 Rehabilitation Research Center, Veterans Administration Connecticut Healthcare System, West Haven, Connecticut, United States of America; 6 Department of Neurology, Yale University School of Medicine, New Haven, Connecticut, United States of America; Sackler Medical School, Tel Aviv University, ISRAEL

## Abstract

During axonal maturation, voltage-gated sodium (Na_v_) channels accumulate at the axon initial segment (AIS) at high concentrations. This localization is necessary for the efficient initiation of action potentials. The mechanisms underlying channel trafficking to the AIS during axonal development have remained elusive due to a lack of Na_v_ reagents suitable for high resolution imaging of channels located specifically on the cell surface. Using an optical pulse-chase approach in combination with a novel Na_v_1.6 construct containing an extracellular biotinylation domain we demonstrate that Na_v_1.6 channels are preferentially inserted into the AIS membrane during neuronal development via direct vesicular trafficking. Single-molecule tracking illustrates that axonal channels are immediately immobilized following delivery, while channels delivered to the soma are often mobile. Neither a Na_v_1.6 channel lacking the ankyrin-binding motif nor a chimeric K_v_2.1 channel containing the Na_v_ ankyrinG-binding domain show preferential AIS insertion. Together these data support a model where ankyrinG-binding is required for preferential Na_v_1.6 insertion into the AIS plasma membrane. In contrast, ankyrinG-binding alone does not confer the preferential delivery of proteins to the AIS.

## Introduction

Voltage-gated sodium (Na_v_) channels are responsible for the initiation and conduction of action potentials in neurons and are densely accumulated at the axon initial segment (AIS) [1]. Na_v_ channels are composed of a highly post-translationally modified pore-forming α-subunit and auxiliary β-subunits [2]. Of the nine Na_v_ alpha subunits (Na_v_1.1–1.9), Na_v_1.1, Na_v_1.2, Na_v_1.3 and Na_v_1.6 are the major isoforms within the central nervous system [3] and the action potential waveform is determined to a large extent by the differential expression and distribution of these isoforms within the somatodendritic and axonal compartments [4–6]. As such, the number, type, and location of channels must be precisely regulated to ensure proper neuronal function.

The AIS is a highly complex structure that can form without glial contact in cultured hippocampal neurons, in contrast to nodes of Ranvier that require interactions with glial cells [7]. The axon can be identified in cultured hippocampal neurons within one to two days in vitro (DIV) and its specialized cytoskeleton and unique protein content develops over the first week in culture [8]. AnkyrinG (ankG), an intermediate anchoring protein that tethers many AIS proteins to the actin-spectrin cytoskeleton, is considered the master organizer of the AIS [9]. AnkG is detected in cultured hippocampal neurons as early as DIV3, increasing in expression levels and becoming highly localized to the AIS by DIV7 [10]. Over a similar timeframe, Na_v_ channels are recruited to the AIS via binding to ankG. This binding is mediated by a sequence of 9 amino acids, the ankyrinG-binding motif (ABM), within the intracellular linker between Na_v_ domains II-III [11, 12], an interaction regulated by the protein kinase CK2 [13, 14].

While the ABM has been shown to be necessary to localize Na_v_1.6 to the AIS [13] the trafficking route taken by these channels as they become concentrated at the AIS during axonal development is still elusive. Initial studies of Na_v_ channel trafficking in neurons used chimeric proteins consisting of a fusion between the extracellular domain of CD4 and the C-terminal cytoplasmic domain of Na_v_1.2 [15]. This construct showed axonal localization. Other CD4 chimeric constructs contained the Na_v_1.2 ABM and accumulated at the AIS following ubiquitous delivery to the cell surface and selective endocytic elimination from the somatodendric regions [16]. The AIS accumulation of this chimeric protein was presumably due to ankG binding. While studies with these chimeras have been indispensable for our understanding of Na_v_ localization and ankG binding, they do not fully recapitulate that of the native channel [13]. In addition to the studies with chimeras, experiments have been performed with GFP-tagged versions of the full length Na_v_1.2 and 1.6 channels in order to address axonal localization [13, 17]. These studies indicated that the Na_v_1.2 N-terminal domain is required for axonal targeting and that the Na_v_1.6 ABM is essential for accumulation at the AIS. However, both the chimera-based studies and those utilizing the GFP-tagged full-length channel could not determine the location of protein delivery to the plasma membrane, rather just the site of steady-state protein accumulation.

In order to address these points, we designed a novel full-length Na_v_1.6 channel construct containing an extracellular biotin tag and intracellular GFP tag. Here we use this construct for imaging studies using sensitive TIRF microscopy to visualize both the site of protein insertion and subsequent membrane dynamics of individual Na_v_1.6 molecules as they are delivered to, and anchored within, the AIS during axonal development. We find that Na_v_1.6 is preferentially inserted into the axonal membrane, where we observe small numbers of channels simultaneously delivered directly to spatially discrete sites. Channels delivered to the axon are immediately immobilized, while channels delivered to the somatodendritic region are often mobile, although they are not observed to diffuse into the AIS. Na_v_1.6 lacking the ABM and a K_v_2.1-Na_v_1.2ABM chimera are not preferentially trafficked to the AIS, demonstrating that ankG binding is necessary for preferential delivery to the AIS.

## Materials and Methods

### Plasmid constructs

The pcDNA3.1-Na_v_1.6 channel was previously rendered TTX–resistant via a Y371S substitution [18]. Overlap PCR mutagenesis techniques were used to introduce KpnI and PacI restriction sites at the C-terminus of Na_v_1.6 for insertion of GFP. Additional sites, NotI and SacII, were introduced between S1545 and K1546 within the extracellular domain between S1-S2 of domain IV, into which a biotin acceptor domain (BAD) was inserted [19]. For the Na_v_1.6-dABM constructs, PCR mutagenesis was used to remove the ankG-binding motif [12], amino acids 1094–1102 (VPIAVGESD). The GFP-K_v_2.1-BAD-Na_v_1.2ABM construct was generated from the HA-K_v_2.1-Na_v_1.2 1,080–1,203-GFP [20] by replacing the ApaI-ApaI fragment from GFP-K_v_2.1-BAD [19]. This exchanged the extracellular HA epitope with the BAD tag. The GFP from ankG-GFP (gift from Vann Bennett, Duke University) was replaced with mCherry using SacII and NotI sites.

### Cell culture and transfections

ND7/23 cells (Sigma-Aldrich, St. Louis, MO, USA) were plated on glass-bottom 35mm dishes (MatTek, Ashland, MA, USA) that had been previously coated with Matrigel (BD Biosciences, San Jose, USA) and cultured in DMEM plus 10% fetal bovine serum supplemented with GlutaMax (Gibco, Life Technologies, Grand Island, NY, USA) and non-essential amino acids (Hyclone, GE Heathcare Life Sciences, Logan, UT, USA). Neuronal cultures were used as previously described [19]. Animal use was according to protocols approved by the Institutional Animal Care and Use Committee of Colorado State University (Animal Welfare Assurance Number: A3572-01). This Institutional Animal Care and Use Committee (IACUC) committee specifically approved this study. Embryonic hippocampal tissue was collected after anesthesia with isoflurane followed by decapitation. Neuronal transfections were performed after days in vitro (DIV)4–6 in culture as indicated for each experiment. Transfections for both cell types were performed with Lipofectamine 2000 (Invitrogen, Life Technologies, Grand Island, NY, USA) and the indicated Na_v_1.6 (1μg) or GFP-K_v_2.1-BAD-Na_v_ABM (0.5μg) channel DNA. When indicated, human β1 in pcDNA3.1Mygro(+), rat β2 in pcDNA3.1VS-HisTopoTA, pSec-BirA (bacterial biotin ligase to biotinylate the Na_v_1.6 or K_v_2.1-Na_v_1.2 proteins [21]), ankG-GFP/mCherry, or pEGFP-C1 (to mark transfected cells) were added. When neurons were used following transfection depended on the specific experiment (DIV of transfection/DIV of experiment): Immunolableing- DIV4 or 6/DIV6,8, or 12 as indicated; surface labeling for AIS to soma ratio and FRAP- DIV4/DIV6,8,10, or 12 as indicated; electrophysiology- DIV4/DIV6; insertion site experiments- DIV4/DIV6 (imaged 36–44 hrs post-transfection for Na_v_1.6 constructs or 12–16 hrs post-transfection for GFP-K_v_2.1-BAD-Na_v_1.2ABM). Endogenous Na_v_1.6 did not appear consistently in our neuronal cultures until DIV10.

### Immunofluorescence

Neurons were fixed in 4% formaldehyde in PBS for 15 min, incubated in PBS + 10% TX-100 + 10% goat serum for 30 min, and labeled with rabbit anti-Na_v_1.6 antibody (Caldwell et al., 2000, 1:100), mouse monoclonal anti-ankyrinG (NeuroMab; 1:1000), or mouse monoclonal anti-MAP2 (Sigma, 1:1000) diluted in PBS containing 10% goat serum and 1% TX-100. Goat anti-rabbit or goat anti-mouse secondary antibodies conjugated to AlexaFluor-488, 594 or 647 (Molecular Probes, Life Technologies, Grand Island, NY, USA) were diluted 1:1000 in 10% goat serum and PBS.

### Fluorescence recovery after photobleaching (FRAP)

Biotinylated Na_v_1.6-BAD-GFP in transfected rat hippocampal neurons (rHNs) were labeled with SA-594 (1:1,000 dilution in imaging saline) at 37°C for 10 min and then washed 3–4 times with imaging saline. Imaging and photobleaching were performed using an Olympus (Tokyo, Japan) FV1000 confocal microscope as previously described [19]. Images were acquired every 1 min and the recovery of both GFP and SA-594 fluorescence within the bleach region quantitated The GFP signals were fit with a single exponential to determine percent recovery. Adjustments to brightness and contrast are identical for comparisons between time points, however, the brightness and contrast were enhanced to allow visualization of the vesicles.

### Detection of intracellular channel insertion into the plasma membrane

Biotinylated Na_v_1.6-BAD-GFP channels on the neuronal surface 36–44 hrs post-transfection (channels do not fully accumulate at AIS until ~48 hrs post-transfection) were blocked using 1 μM NeutrAvidin for 5 min, then washed 5–6 times with imaging saline. During TIRF imaging (2.5 Hz, 100 ms exposure), 1 nM SA-594 was added to the bath. Controls for the effectiveness of NeutrAvidin block, necessity of channel trafficking for binding, and kinetics of fluorophore binding were performed as previously reported [22].

### Electrophysiology

Transfected ND7/23 cells were patch-clamped 24–48 hrs after transfection in a neuronal external solution consisting of (in mM): 110 NaCl, 20 TEA-Cl, 2 MgCl_2_, 1.2 NaH_2_PO_4_, 11.1 glucose, and 10 HEPES, pH 7.4 (adjusted with NaOH), 301 mOsm. 300 nM TTX was added to the bath. The pipette solution contained (in mM): 125 CsCl, 10 NaCl, 1.5 MgCl, 5 HEPES, 0.5 EGTA, pH 7.4 (adjusted with CsOH), 278 mOsm. Pipettes had resistances of 1.4–2.3 MΩ and R_a_ was 5 MΩ or less. Whole-cell Na^+^ currents were recorded at room temperature using an Axopatch 200B amplifier (Molecular Devices, Sunnyvale, CA, USA). Ionic currents were capacitance and series resistance compensated by 70–90%, sampled at 10 kHz (Digidata 1440; Molecular Devices, Sunnyvale, CA, USA), filtered at 2kHz, and leak subtracted online using the P/4 method in pClamp10. Cells were held at -80 mV, stepped to -120 mV for 50 ms to release channels from inactivation, then depolarized to potentials between -70 and +75 mV for 100 ms in 5 mV steps. Activation curves were plotted by converting peak ionic currents (I) to conductance (G) using the relationship: G = I/(V—E_Na_). E_Na_ was calculated to be +60 mV. Steady-state inactivation was determined using a 100 ms prepulse ranging from -140 to 40 mV (10mV increments), then stepping to a potential of -10 mV for 40 ms to measure the non-inactivated channels. Peak inward currents were then normalized to the maximal peak current (I_max_). Curves were fit with a Boltzmann: y = A2 + (A1—A2)/ (1+exp[(V—V_1/2_)/k] where A1 is the initial value, A2 is the final value, V is the test potential, V_1/2_ is the voltage midpoint, and k is the slope factor. Whole cell currents from transfected neurons (DIV6, 2 days post-transfection) were measured as described above.

### Image acquisition, presentation, and data analysis

Neurons expressing fluorescent protein–tagged constructs were imaged with either an Olympus FV1000 confocal or a Nikon TIRF microscope as previously described [22]. Volocity 6.1.1 (PerkinElmer, Waltham, MA, USA) software was used to determine fluorescent intensities, create the kymograph, adjust contrast, and quantitate fluorescence. For the surface density ratios, the AIS and soma ROIs were automatically detected using the object finder in Volocity. The ImageJ Manual Tracking plugin was used to track individual SA-594 molecules. OriginPro 8.5 (OriginLab, Northampton, MA, USA) was used to analyze and fit numerical data. All measurements of fluorescence intensities were background subtracted from an ROI of similar size to the region being measured. For the ratio of surface density of AIS to somatic Na_v_1.6, signal in the soma was often only moderately above that of background. For these experiments, background was determined by taking the mean of two background ROI’s. Images presented contain identical adjustments for brightness and contrast except where noted. Detection of insertion events, visualized as appearance of SA-594, was through manual inspection. Particles that appeared but remained for less than 1 s were considered non-specific and were excluded. Diffusion coefficients were calculated from the mean square displacements as previously described [21]. For all imaging experiments, the stage and objective were heated to 37°C.

### Statistics

Data are presented as mean ± s.d. unless otherwise stated. Statistical analysis was performed in Sigmaplot (Systat Software, Inc., San Jose, CA, USA) using an unpaired t-test, paired t-test, or a Mann-Whitney Rank Sum Test for non-parametric data. p<0.05 was considered significant.

## Results

### GFP and biotinylation domain-tagged Na_v_1.6 channels localize to the axon initial segment in cultured hippocampal neurons

**Fig 1:**
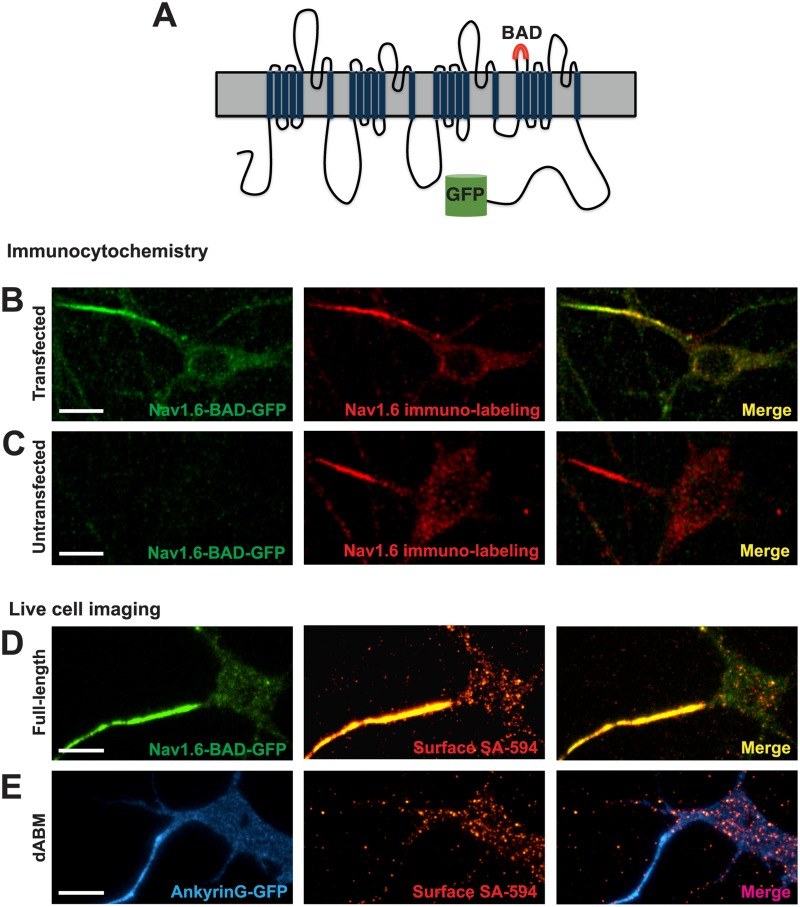
Na_v_1.6-BAD-GFP expression mimics that of the endogenous channel in cultured rat hippocampal neurons. **A)** Schematic of the Na_v_1.6-BAD-GFP construct showing location of the biotin acceptor domain (BAD) and fusion of GFP to the channel C-terminus. (**B-C)** Comparison of anti-Na_v_1.6 immunolabeling in transfected and untransfected hippocampal neurons. Compressed confocal z-stack of GFP fluorescence (left panel) and anti-Na_v_1.6 immunolabeling (middle panel) from a Na_v_1.6-BAD-GFP transfected neuron **(B)** or a non-transfected neuron **(C)**. Overlay (right panel). Fluorescence intensity of immunolabeling for transfected vs endogenous AIS Na_v_1.6 expression was not significant (t-test, p = 0.132). **(D-E)** Surface expression of full-length Na_v_1.6 or Na_v_1.6 lacking the ankG-binding motif (ABM) in live neurons visualized by TIRF microscopy. **D)** Na_v_1.6-BAD-GFP is highly enriched at the AIS as indicated both by total expressed protein (green) and surface expression (red) visualized by live cell labeling with SA-594. **E)** Na_v_1.6-dABM-BAD co-expressed with ankG-GFP (blue) as an axonal marker. Surface channels labeled with SA-594 (red) demonstrated a lack of axonal localization while maintaining a somatic expression pattern similar to the full-length channel. Panels B and C are taken from DIV12 cultures since the endogenous Na_v_1.6 does not appear until this developmental time. Panels D and E are from DIV8 cultures. Scale bars represent 10 μm.

To enable real-time imaging of Na_v_1.6 dynamics in neurons, GFP was fused to the C-terminus of the mouse Na_v_1.6 channel and a biotin acceptor domain (BAD) (Tamkun et al., 2007) was inserted into the S1-S2 extracellular loop of domainIV (Fig 1A). The S1-S2 linker was chosen due to the lack of sequence conservation between isoforms as well as no known role in neurotoxin binding or channel function [23]. The BAD is recognized by a bacterial biotin ligase that is co-transfected with the channel. This ligase biotinylates a lysine residue in the center of the inserted 17 amino acid sequence, allowing for specific labeling of surface channels via streptavidin-conjugated fluorophores [24].

The GFP and BAD-tagged Na_v_1.6 channel (Na_v_1.6-BAD-GFP) was expressed in cultured rat hippocampal neurons (rHN) and visualized via confocal and TIRF microscopy. To confirm that the expression levels and localization of the transfected Na_v_1.6 resembled that of the endogenous Na_v_1.6, immunolocalization studies were performed using an antibody specific for Na_v_1.6 [25]. Fig 1B shows a compressed confocal z-stack of the GFP fluorescence (left panel) and antibody labeling (middle panel) observed in DIV12 rHN cultures 6 days post-transfection. This time was chosen since the endogenous Na_v_1.6 channel does not appear until DIV10. Fig 1C shows the antibody labeling of endogenous Na_v_1.6 (middle panel). The anti-Na_v_1.6 immuno-labeling intensity levels are nearly identical between the transfected and non-transfected neurons in this example, and for the population of transfected neurons we detected only 1.3-fold more Na_v_1.6 at the AIS, 501±321 arbitrary units (a.u.) (n = 18), than that found endogenously, 375±204 a.u. (n = 14) (ns;*t*-test), minimizing concerns about artifacts due to overexpression of the recombinant channel. Since Na_v_1.6 has been reported to localize to the distal region of the AIS [5], we compared the distribution of transfected Na_v_1.6-BAD to the endogenous channel within this region. Immunolocalized Na_v_1.6 was concentrated in the distal region of the AIS as defined by ankG immunolabeling in DIV12 rHNs for both the transfected and endogenous channels. However, for DIV6 rHNs the transfected channel was concentrated in the distal AIS in only half of the cells examined. This variability is not surprising since the AIS is changing rapidly during this developmental period. Expression of the Na_v_1.6-BAD did not alter AIS length, for the immunolocalized ankG averaged 18.1±4.0 and 17.3±4.7 μm (n = 9 and 8, ns; *t*-test, p = 0.7) for DIV12 neurons expressing the transfected and endogenous channel, respectively.

Since GFP fluorescence does not reliably report true surface expression, the surface distribution was instead visualized via live-cell labeling of the extracellular BAD tag with streptavidin-conjugated AlexaFluor-594 (SA-594) (Fig 1D). The surface-specific fluorescence derived from the SA-594 (Fig 1D, middle panel) indicates robust surface localization at the AIS. Interestingly, surface Na_v_1.6 labeling showed a punctate localization pattern on the soma. The ratio of surface density for channels in the AIS plasma membrane compared to the soma surface as visualized by fluorescence intensity of the surface label remained relatively constant with time in culture post-transfection (38±6, n = 11; 33±8, n = 11; 32±4, n = 16; 38±7, n = 13 for DIV6, 8, 10, and 12 respectively; ns;anova).

The extracellular BAD tag was also inserted into a Na_v_1.6 construct lacking the critical amino acids within the ankG-binding motif needed for ankG binding, amino acids 1094–1102 [12] (Na_v_1.6-dABM-BAD). Consistent with published data [13], this channel showed no AIS accumulation with the SA-594 surface fluorescence evenly distributed throughout the soma and AIS, as marked by ankG-GFP (Fig 1E). However, the puncta on the soma were still present indicating that an ankG-independent mechanism localizes Na_v_1.6 channels on the soma surface (Fig 1E, middle panel).

**Fig 2:**
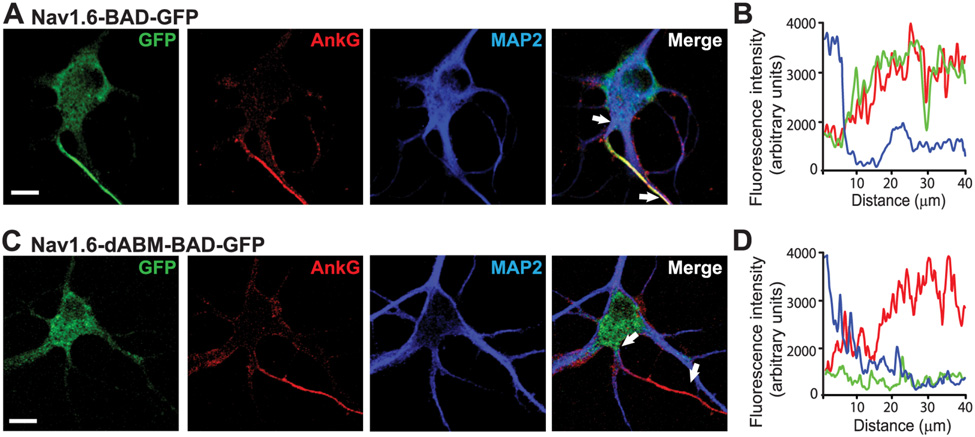
Na_v_1.6-BAD-GFP but not Na_v_1.6-dABM-BAD-GFP co-localizes with axonal markers. **(A,C)** Compression of confocal z-stacks of DIV8 rat hippocampal neurons co-transfected with ankG-mCherry and either wild-type Na_v_1.6-BAD-GFP or Na_v_1.6-dABM-BAD-GFP and immunolabled for MAP2. **A)** Na_v_1.6 is enriched within the ankG positive AIS. **B)** Line profile of the AIS showing the simultaneous increase of Na_v_1.6 (green) and ankG (red) and the decrease of MAP2 (blue) with increasing distance from the soma. **C)** Na_v_1.6-dABM-BAD-GPF is found throughout the somatodendritic region of the neuron, but is not enriched in the ankG positive AIS. **D)** Line profile of the AIS showing the increase in ankG (red) while the expression levels of the mutant Na_v_1.6 (green) and MAP2 (blue) are low within the AIS. Scale bars represent 10 μm.

In order to establish the distribution of these Na_v_1.6 constructs relative to the AIS we performed colocalization studies with ankG and MAP2 as illustrated in Fig 2. AnkG concentrates at the AIS and is viewed as the AIS organizer [9, 26], while MAP2 is found in dendritic processes and is dramatically reduced in the axon [13]. Na_v_1.6-BAD-GFP channels clearly associate with the ankG positive and MAP2 negative AIS (Fig 2A and 2B), while the Na_v_1.6-dABM-BAD-GFP mutant does not (Fig 2C and 2D).

### GFP- and biotinylation domain-tagged Na_v_1.6 channels demonstrate functional properties very similar to the wild-type channel

**Fig 3:**
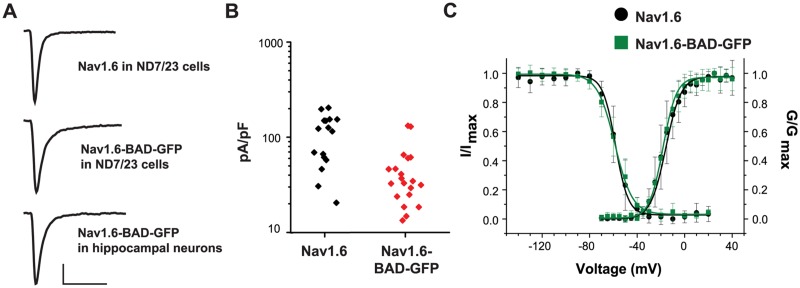
Na_v_1.6-BAD-GFP displays wild-type currents. ND7/23 cells or DIV6 rat hippocampal neurons were transfected with either the wild-type channel or Na_v_1.6-BAD-GFP and the resulting TTX-resistant currents analyzed by whole-cell voltage-clamp in the presence of 300 nM TTX. **A)** Representative current traces from the indicated cell type and construct. Scale bars represent 500 pA and 5 ms. B) Summary of current densities for WT and Na_v_1.6-BAD-GFP expressed in hippocampal neurons. The difference in mean peak current density (116.8±63.06, n = 16 and 48.42±35.73 pA/pF, n = 20,) was significant (p<0.001) although many of the cells expressing the Na_v_1.6-BAD-GFP channel had expression levels similar to cells expressing the wild-type channel. Currents were recorded 2 days post-transfection. **C)** Voltage-dependence of fast-inactivation and activation for wild-type Na_v_1.6 (•) and Na_v_1.6-BAD-GFP (∎) as measured in ND cells 36–48 hours post-transfection. Error bars are mean ± s.d.

Na_v_1.6 has previously been tagged with GFP on the channel C-terminus with retention of wild-type activity [13]. To verify that the insertion of the BAD had minimal effects on function, we transfected the wild-type and Na_v_1.6-BAD-GFP constructs into both the ND7/23 neuronal cell line and rHNs and analyzed channel properties via whole-cell voltage-clamp recordings. The Na_v_1.6 channel was previously rendered tetrodotoxin (TTX)-resistant (Y371S), which allows easy isolation of the transfected Na_v_1.6 current by blocking the endogenous TTX-sensitive inward currents with 300nM TTX [18]. Fig 3A illustrates that similar current waveforms were obtained when comparing the wild-type and Na_v_1.6-BAD-GFP constructs in ND7/23 cells and hippocampal neurons. Current densities for the transfected wild-type Na_v_1.6 and the Na_v_1.6-BAD-GFP in rHN averaged 116.8±63.06, n = 16 and 48.42±35.73 pA/pF, n = 20, respectively. While the differences in mean current densities are significant (p<0.001; Mann-Whitney), the distribution illustrated in Fig 3B illustrates that there is considerable overlap. The endogenous Na_v_ currents in DIV6 non-transfected neurons as measured in the absence of TTX, presumably carried by a mixture of Na_v_1.1, Na_v_1.2, and Na_v_1.6 [27], had a current density of 214.3±64.9 pA/pF, n = 6. The current density of the recombinant Na_v_1.6 channels (48–117 pA/pF) is in agreement with the endogenous Na_v_1.6 channel contribution of 40% to the TTX-S current in hippocampal neurons [28]. Fig 3C shows the voltage-dependence of activation and fast-inactivation for the wild-type and Na_v_1.6-BAD-GFP constructs following expression in ND cells. The wild-type and tagged channels showed activation midpoints of -15.8±0.8, n = 6 and -18.3±0.4 mV, n = 8, and fast-inactivation midpoints of -58.2±1.3, n = 6, and -58.7±0.1 mV, n = 9, respectively, (values are mean ± s.e.m.) which are in agreement with published values [13, 29]. The rate of fast inactivation was comparable between the wild type and Na_v_1.6-BAD-GFP constructs, with time constants of (0.78±0.03, n = 6; 1.00±0.05, n = 7; mean ± s.e.m.), respectively. This difference does not achieve statistical significance (p = 0.051, Mann-Whitney).

Together, Figs 1–3 indicate that the Na_v_1.6-BAD-GFP channels have expression patterns and functional properties very similar to the endogenous Na_v_1.6 and thus are suitable for trafficking studies in rHNs.

### Na_v_1.6-BAD-GFP channels are localized to the AIS via direct insertion

**Fig 4:**
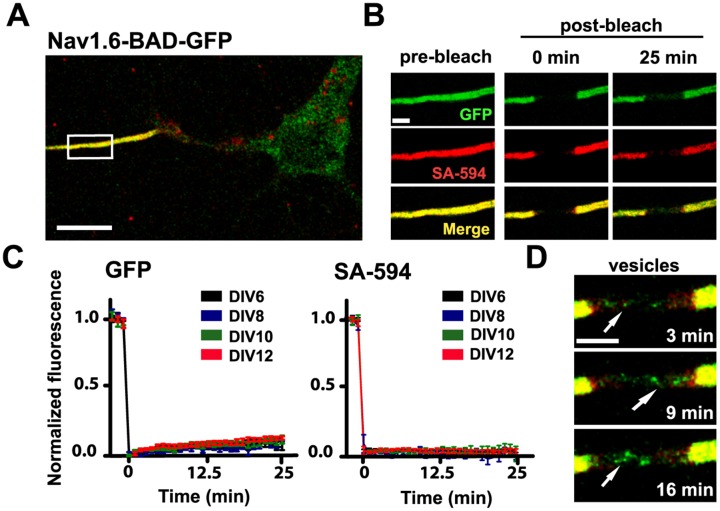
Na_v_1.6 is stable in the AIS of mature neurons. **A)** A representative compressed confocal z-stack of a DIV10 rHN expressing Na_v_1.6-BAD-GFP. The high density of Na_v_1.6 in the AIS is labeled both by the GFP fluorescence (green) and by surface labeling of the BAD tag via SA-594 (red). **B)** Enlargement of the white box in (**A**) showing fluorescence before photobleaching, immediately after photobleaching and 25 min postbleach. **C)** Average normalized FRAP curves over 25 min for the GFP fluorescence and surface-specific SA-594 fluorescence. Days post-transfection are indicated. On average, the GFP recovered 7.5±0.2%, n = 8, for DIV6 and 9.7±1.8%, n = 6, for DIV10 mean ± s.e.m.)Images were acquired every minute to minimize photobleaching during the recovery. D) Detection of mobile GFP-containing trafficking vesicles within the bleached AIS. The different time points illustrate the detection of dynamic puncta (arrows) at the indicated postbleach times. Scale bars represent 10 μm (A) or 2 μm (B,D).

As indicated in Fig 4, once Na_v_1.6-BAD-GFP channels have accumulated within the AIS of cultured hippocampal neurons channel turnover is slow. The illustrated FRAP studies of both cell surface and total Na_v_1.6-BAD-GFP within the AIS indicates that there is only approximately 10% recovery of the GFP fluorescence over 25 min (7.5±0.2%, n = 8 for DIV6; 9.7±1.8%, n = 6 for DIV10; mean ± s.e.m.) while there is no detectable recovery of the surface-specific SA-594 fluorescence. Such limited recovery complicates study of AIS delivery in neurons where Na_v_1.6 accumulation has reached steady state. For this reason we focused our insertion site experiments on the delivery of nascent channels to the developing AIS in DIV6 neurons. Interestingly, we often observed mobile puncta, likely representing trafficking vesicles moving through the bleached region as illustrated in Fig 4D.

**Fig 5:**
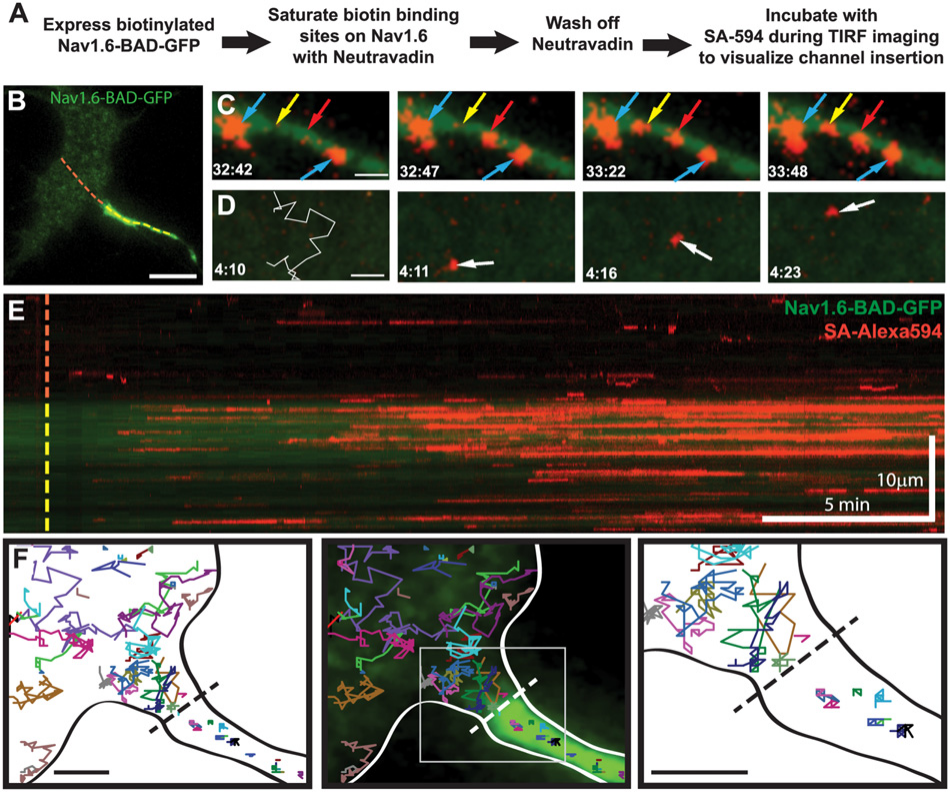
Na_v_1.6-BAD-GFP is delivered along the length of the AIS where it is immediately immobilized. **A)** Experimental outline. **B)** Representative TIRF image and reference for the Kymograph in (**E**). Dotted line indicates line scan starting in the soma (red) and continuing through the AIS (yellow). **C)** Representative images of SA-594 labeling of newly inserted channels in the AIS. Two SA-594 puncta appear in the axonal membrane previously devoid of surface labeling (yellow and red arrows), denoting insertion events. Two other puncta are present throughout this time course and show no lateral movement (blue arrows). **D)** Representative images of SA-594 labeling of newly inserted channels in the soma. The left panel shows the insertion site location (O) and subsequent single-molecule track overlaid on the region prior to the channel delivery at 4:11. **E)** Kymograph of the line scan shown in (**B**) indicating GFP (green) and SA-594 (red) fluorescence over time. **F)** Single-molecule tracking of inserted Na_v_1.6-BAD-GFP. Each colored line represents an individual track. The black dotted line indicates the soma/AIS boundary. Neurons were transfected on DIV4 and imaged 36–44 hrs post-transfection. Scale bars represent 10 μm (B,E) or 2 μm (C,D,F).

Fig 5A outlines the experimental approach for an optical pulse-chase experiment used to detect cell surface insertion sites. Briefly, NeutrAvidin was used to saturate available biotin binding sites on Na_v_1.6-BAD-GFP channels already present on the neuronal surface. The unbound NeutrAvidin was then removed and SA-594 added to the bath during imaging. Thus, the spontaneous appearance of SA-594 fluorescence indicates a newly inserted channel. The advantage here is that the location of even a single newly inserted channel can be mapped since 1) our TIRF microscope readily detects single 594-SA molecules and 2) SA binding is rapid (T_1/2_ < 75 sec) compared to the rate of membrane protein diffusion [22].

Examples of delivery to both the soma and AIS are shown in Fig 5C and 5D, respectively. The left panel of Fig 5C shows a region of AIS membrane with two isolated puncta of SA-594 labeling (blue arrows) and the rest of the membrane devoid of SA-594 fluorescence. After 5 (yellow arrow) and then 35 (red arrow) seconds, new SA-594 puncta appear spontaneously. All puncta persist in the same location after their appearance. Both the spontaneous appearance of labeled channels and their immediate stability support the idea that Na_v_1.6 channels are inserted directly into the AIS via direct trafficking. In contrast, SA-594 fluorescence that spontaneously appeared on the soma was often followed by rapid diffusion away from the site of channel insertion (Fig 5D). The white trace (Fig 5D, left panel) illustrates the single-molecule diffusion track over 13 sec after insertion at the red circle.

To visually represent the insertion events within the soma and AIS throughout the image sequence, a kymograph was created (Fig 5E) corresponding to the line-scan indicated by the dashed line in Fig 5B. The appearance of SA-594 molecules is seen along the length of the AIS, denoting insertion events. These particles persist over time as horizontal lines in the kymograph due to limited lateral movement, suggesting that they are immediately immobilized after insertion. Had channels been localized to the AIS via diffusion from the soma, lines with a negative slope would have been obvious in the kymograph. Insertion events were seen in both the soma and AIS; however, there was a strong preference for the AIS. Out of 5 cells analyzed the number of insertion events in the AIS per μm^2^ was 0.79±0.11, while the insertions in the soma were only 0.11±0.04 (mean±s.e.m.). Channels inserted into the somatic region were either mobile, as demonstrated by a brief appearance on the kymograph, or static and thus generating persistent hortizontal lines. These behaviors are consistent with some somatic channels localizing to small puncta as observed in Fig 1D.

While these data demonstrate that Na_v_1.6 localizes to the AIS primarily through direct insertion, this does not exclude the possibility of diffusion trapping, i.e. mobile channels being trapped via ankyrinG binding once entering the AIS, especially since we used DIV6 rHNs and the soma/AIS diffusion barrier is not well established until DIV10 [30]. To address this, we used single molecule tracking of newly inserted channels to inspect Na_v_1.6 movement in the vicinity of the AIS/soma interface. Fig 5F shows representative tracks from throughout the imaging sequence, demonstrating that while channels approached the AIS, none crossed the boundary. The diffusion coefficient for the mobile channels on the soma as calculated from mean square displacement analysis was 0.07 ±0.05 μm^2^/s, n = 20, in close agreement with previously determined values for mobile membrane proteins [21]. The channels in the AIS membrane had diffusion coefficients of 0.0007±0.0006 μm^2^/s, n = 20, similar to previous reports of Na_v_ channels [30] or K_v_2.1-Na_v_ chimeras [20] in the AIS. In summary, the data presented in Fig 5 argue for preferential delivery to the AIS and immediate binding to ankG, with no direct evidence of diffusion trapping.

### Insertion events simultaneously deliver multiple Na_v_1.6 channels to the AIS

**Fig 6:**
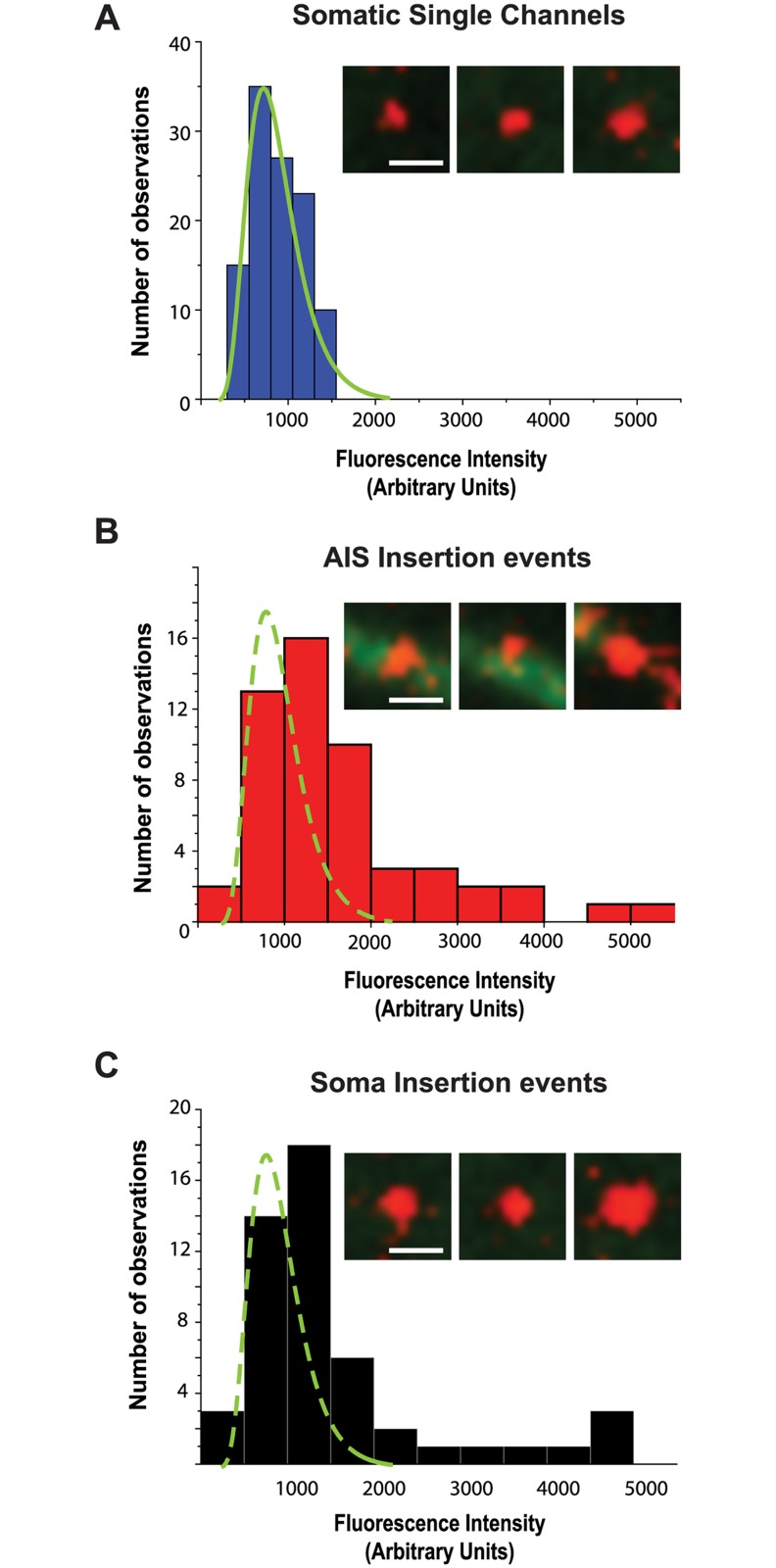
Simultaneous insertion of multiple channels into the plasma membrane. **A)** Histogram of fluorescence intensity of SA-594 labeled Na_v_1.6-BAD-GFP channels on the soma. Solid green line is a fit to the histogram distribution. (**B-C)** Fluorescence intensity of delivered channels in the AIS and soma, respectively. Dashed green line is the fit to the histogram distribution from panel A. Representative images are shown in the insets. Scale bars represent 1 μm.

During the FRAP studies illustrated in Fig 4D puncta of GFP fluorescence were often observed moving through the bleached AIS. These likely represent trafficking vesicles although we cannot conclude here that these represent cargo on the way to the cell surface. However, in the TIRF-based insertion site experiment the simultaneous appearance of multiple channels, which should be detected as multiple SA-594 molecules binding within the same delivery site, would be indicative of vesicular delivery to the plasma membrane. To investigate this, we first ascertained the average fluorescence intensity of single SA-594 labeled channels within the soma, identified by their distinctive, diffusive behavior in the plasma membrane (Fig 5D). This produced a distribution with a mean of 863.3±295.7 a.u., n = 110 (Fig 6A). In contrast, puncta spontaneously appearing on the plasma membrane of the AIS and soma had fluorescence intensities as much as 6-fold greater than that of single molecules (Fig 6B and 6C), with mean values of 1615.5±1030.7 a.u., n = 53 and 1560.9±1134.3 a.u, n = 50, respectively. These data are consistent with multiple SA-594 molecules binding rapidly following new channel insertion, with up to 6 channels per insertion event.

### Preferential AIS delivery requires ankG binding

**Fig 7:**
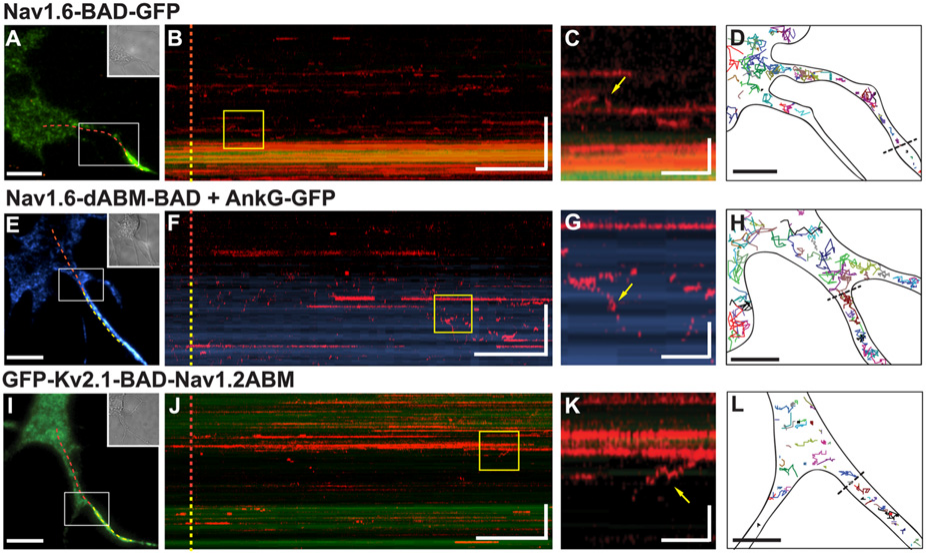
AnkyrinG binding is necessary to confer directed delivery to the AIS. Insertion site experiments were performed as outlined in Fig 4A for hippocampal neurons transfected with either full-length Na_v_1.6-BAD-GFP **(A-D)**, Na_v_1.6-dABM-BAD and ankG-GFP **(E-H)**, or GFP-K_v_2.1-BAD-Na_v_1.2ABM **(I-L)**. **A,E,I)** Reference images for the kymographs with the dotted line indicating the line scan starting in the soma (red) and continuing through the AIS. **B,F,J)** Kymographs of line scans indicating location of newly inserted channels over time as indicated by SA-594. **C,G,K)** Enlargements of yellow boxes in kymographs. Immobile channels appear as persistent horizontal lines, while mobile channels are represented by the vertical movements (yellow arrows) in the kymograph or brief appearances as channels cross the line scan. **D,H,L)** Representative single molecule tracks illustrating channel mobility. The black dotted lines indicate the soma/AIS boundary. Neurons were transfected on DIV4 and imaged 36–44 hrs post-transfection for the Na_v_1.6 constructs or 12–15 hrs post-transfection for the GFP-K_v_2.1-BAD-Na_v_1.2ABM construct. Scale bars for **A,E,I** represent 10 μm. Scale bars for **B,F,J** represent 2 min (horizontal) and 10 μm (vertical). Scale bars for **C,G,K** represent 15 sec (horizontal) and 3 μm (vertical). Scale bars for **D,H,L** represent 5 μm.

In order to address the role of ankG binding in the delivery of Na_v_1.6 to the AIS, we repeated the insertion site assay (Fig 5A) using the Na_v_1.6 construct lacking the ankG-binding motif (Na_v_1.6-dABM-BAD). To facilitate comparison, another insertion kymograph for the full-length Na_v_1.6-BAD-GFP channel is presented in Fig 7A–7C. Similar to the kymograph in Fig 5, Na_v_1.6 insertion is most prominent in the AIS, with some SA-594 labeled channels present in the soma. Panel C shows an enlargement of the boxed region in the kymograph which highlights channel mobility on the soma as demonstrated by the vertical movement of the SA-594 signal (yellow arrow). This mobility is also apparent in the single molecule tracks of Panel D. Note that a short-lived SA-594 signal on the kymograph does not distinguish between channel movement, photobleaching, or internalization. In contrast to the full-length Na_v_1.6, the kymograph illustrating surface delivery of the Na_v_1.6-dABM-BAD mutant channel (Panel F) demonstrates that the preferential AIS insertion is lost upon deletion of ankG binding activity. Here the neurons were also transfected with ankG-GFP to mark the AIS. Overall, insertion events for the Na_v_1.6-dABM-BAD channels were 0.18±0.01 per μm^2^ in the AIS and 0.11±0.01 per μm^2^ (mean±s.e.m.) in the soma. The corresponding single molecule tracks shown in Fig 7H emphasize that the Na_v_1.6-dABM-BAD mutant has increased mobility within the AIS as compared to the channel with ankG binding capability. The calculated diffusion coefficients from the mean square displacements were 0.09±0.06 μm^2^/s (n = 10) for somatic channels and 0.03±0.03 μm^2^/s (n = 10) for channels in the AIS. The reduced mobility within the AIS relative to the soma for this ankG binding deficient channel is not surprising given the dense cortical cytoskeleton within the AIS [31].

**Fig 8:**
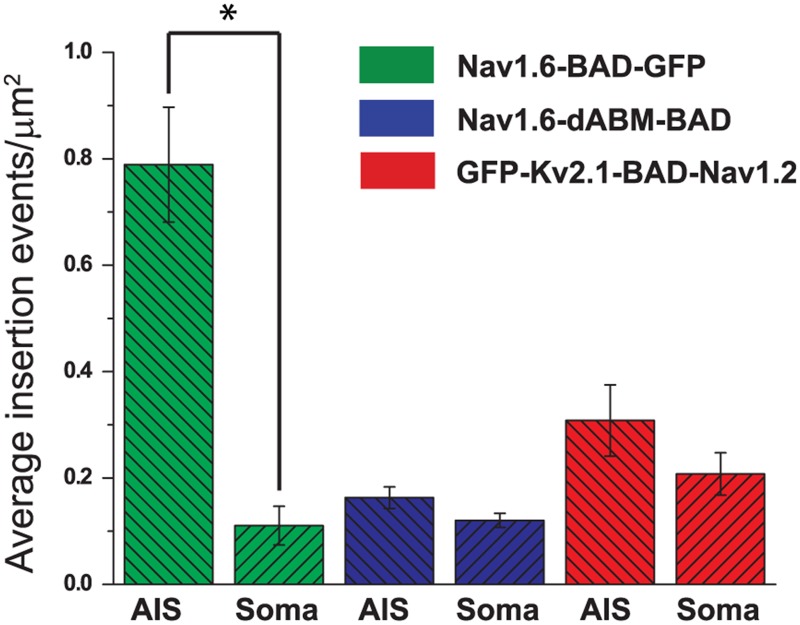
Summary of insertion events for the AIS versus soma. Average insertion events for full-length Na_v_1.6 (694 insertion events from 5 cells), Na_v_1.6-dABM (265 insertion events from 5 cells), or the K_v_2.1-Na_v_1.2 chimera (618 insertion events from 5 cells). Numbers of insertion events were determined over a 5 min period for each region of the cell, then normalized to the surface area. Data shown as mean values ± s.e.m. Significance was determined using paired t-tests. * indicates p = 0.00194 for Na_v_1.6-BAD-GFP. The differences for GFP-K_v_2.1-BAD-Na_v_1.2 were not significant, p = 0.2.

To assess whether the ankG binding domain alone is sufficient for preferential insertion in the AIS, we repeated the insertion site experiment with the K_v_2.1-Na_v_ABM chimera used previously by the Dargent group [14, 20]. These investigators demonstrated that this construct accumulates at the AIS due to its ankG binding activity. We inserted the BAD tag into the S1-S2 extracellular domain of K_v_2.1 to create GFP-K_v_2.1-BAD-Na_v_1.2ABM (GFP-K_v_2.1-BAD-Na_v_1.2). As shown in Fig 7I–7K, the GFP-K_v_2.1-BAD-Na_v_1.2 chimera also lacks preferential insertion into the AIS and is robustly delivered to the cell surface, consistent with what was observed for the CD4-Na_v_II-III chimera [16]. Overall, insertion events per μm^2^ were 0.31±0.06 in the AIS and 0.2±0.03 (mean±s.e.m.) in the soma. Both the single molecule tracks shown in Fig 7L and the diffusion coefficients, 0.018±0.016 μm^2^/s (n = 10) on the soma and 0.0025±0.0026 μm^2^/s (n = 10) within the AIS, indicate that GFP-K_v_2.1-BAD-Na_v_1.2 has 10-fold lower mobility in the AIS as compared to the soma as previously described [20]. The data represented in Fig 7 are summarized in Fig 8.

## Discussion

In this study we used a novel Na_v_1.6 construct tagged with both GFP and an extracellular biotinylation domain to study the membrane delivery of the full-length Na_v_1.6 protein during AIS development. The novelty of this construct lies in the ability to observe the total population of expressed channels via the GFP tag, while also being able to selectively label the subpopulation of surface channels via the biotinylated BAD tag. As summarized in Fig 8 we found channel delivery to the plasma membrane directly within the AIS at a significantly higher density than in the somatodendritic region of the developing hippocampal neurons used in this study. Additionally, individually labeled channels showed almost no diffusion within the AIS after insertion, presumably due to immediate binding to ankG and the associated cytoskeletal elements. Consistent with the idea of vesicular delivery of channels, we observed trafficking vesicles in the FRAP experiments and, in the insertion site experiments, the appearance of newly delivered puncta on the AIS with fluorescence intensities as much as 6-fold greater than that of a single channel. Our data are best explained by delivery of small quanta of channels to spatially discrete sites within the cell membrane at the AIS.

This preferential delivery is dependent on the ability of Na_v_1.6 to bind ankG, as supported by our results from the Na_v_1.6-dABM-BAD mutant. This finding agrees with the recent published results [32] demonstrating that ankG links Na_v_ channels to kinesin-1 and is essential for axonal targeting both in culture and *in vivo*. It appears that ankG binding is only required for preferential delivery and not general trafficking, since non-selective delivery of the Na_v_1.6-dABM-BAD channels to the cell surface was still observed.

As evidenced by the GFP-K_v_2.1-BAD-Na_v_1.2 chimera, ankG binding alone, while required for directed delivery of Na_v_1.6 to the AIS, is not sufficient for preferential delivery to the AIS. Despite the lack of preferential delivery, this chimera is ultimately concentrated at the AIS [20] and thus must use a different mechanism, such as selective endocytosis or diffusion trapping. Previous studies addressing Na_v_ localization mechanisms used a similar chimeric protein, CD4-Na_v_II-III chimera [16]. This construct also appeared on the soma surface only to be internalized within 20 min. These results led to a model in which Na_v_ channels are selectively internalized from the surface if they are not retained via ankG binding at the AIS (for a recent review see [33]). This model is consistent with our GFP-K_v_2.1-BAD-Na_v_1.2 chimera insertion data where this protein is inserted ubiquitously and then presumably endocytosed from the soma but retained in the AIS.

While our data suggest that the majority of the full-length Na_v_1.6 channels arrive at the AIS via direct delivery, we were not able to address the possible contribution of selective endocytosis in enhancing their polarized distribution. Additionally, while it is likely that we were detecting the appearance of nascent channels on the plasma membrane, we were not able to directly address transcytosis in which channels are first inserted into the somatodendritic region, then endocytosed and subsequently delivered to the AIS. Future studies will investigate these mechanisms, as well as further examining the motors and exocytic machinery responsible for vesicular delivery.

Our study focuses on the establishment of the AIS, rather than its maintenance for several reasons. First, it is important to understand how Na_v_1.6 channels are incorporated into the unique cytoskeletal and membrane network created in this region during its development. Additionally, once Na_v_1.6 accumulates at the AIS turnover is very slow (10% over 25 min) as illustrated in Fig 4. Consistent with the immediate immobilization of individual channels delivered to the AIS as illustrated in Fig 5, the lack of surface channel (SA-594 fluorescence) recovery suggests that once Na_v_1.6 is added to the surface it is anchored in place, showing little exchange with adjacent membrane. Thus, while our insertion site assay is useful for detecting the large bolus of Na_v_1.6 channel delivered to the AIS during neuronal development, i.e. how the first wave of Na_v_1.6 populates the AIS, new assays must be developed to be able to detect the much more subtle process of Na_v_1.6 turnover during AIS maintenance. It is interesting to note, however, that even in the absence of a fully established diffusion barrier Na_v_1.6 was not observed to localize to the AIS via diffusion trapping. This strongly suggests that the same trafficking method is used for mature neurons. Whether these results apply *in vivo* will require new technologies as present approaches for the mapping of cell surface delivery sites require a cell system suitable for high-resolution microscopy.

Our present study does not address the role of Na^+^ channel beta subunits in Na_v_1.6 delivery to the AIS, for the beta1 and beta2 subunits were transfected with the Na_v_1.6 to ensure that they were always available. The role of beta subunits in the preferential delivery to the AIS clearly merits study since beta1 binds AnkG [34] and beta1 null mice show no Na_v_1.6 at the AIS even though Na_v_1.1 is up-regulated here [35]. It is possible that a role for beta subunits might explain the lack of K_v_2.1ABM construct delivery to the AIS.

Another unresolved question is related to the nanoscopic organization of the cytoskeleton at the AIS plasma membrane. A recent study elegantly examined actin and spectrin structure at the AIS with super-resolution techniques [31]. Actin rings approximately 100 nm apart (seen in DIV5-7 neurons) are linked by a dense spectrin mesh that at first glance should block even 50 nm vesicles from reaching the cell surface. The most parsimonious explanation to reconcile the presence of a rigid structure and directed AIS delivery is to suggest that the AIS cytoskeleton is dynamic, with transient and localized breakdown in this cytoskeletal collar allowing exocytosis. However, it is noteworthy that platinum replica electron microscopy [36] has not detected dense actin meshworks in the AIS, underscoring the need for further study.

Direct vesicular delivery at the AIS provides a mechanism that can effectively establish sodium channel localization within this critical neuronal compartment. This mechanism of localized exocytosis avoids the soma/AIS plasma membrane diffusion barrier. In addition, a diffusion-trap mechanism is unreliable given the dense membrane and cytoskeletal architecture within the AIS itself [31], especially when considering the cytoplasmic mass of Na_v_ channels and potential association with cytosolic proteins. Direct delivery of Na_v_ channels to the AIS is a means of precisely controlling number, isoform, and location, and thus, the establishment of the proper environment for action potential initiation.
